# Genomic prediction for agronomic traits in a diverse Flax (*Linum usitatissimum* L.) germplasm collection

**DOI:** 10.1038/s41598-024-53462-w

**Published:** 2024-02-08

**Authors:** Ahasanul Hoque, James V. Anderson, Mukhlesur Rahman

**Affiliations:** 1 Department of Plant Sciences, North Dakota State University, Fargo, ND USA; 2 Department of Genetics and Plant Breeding, Bangladesh Agricultural University, Mymensingh, 2202 Bangladesh; 3 USDA-ARS, Edward T. Schafer Agricultural Research Center, Fargo, ND USA

**Keywords:** Genetics, Plant sciences

## Abstract

Breeding programs require exhaustive phenotyping of germplasms, which is time-demanding and expensive. Genomic prediction helps breeders harness the diversity of any collection to bypass phenotyping. Here, we examined the genomic prediction’s potential for seed yield and nine agronomic traits using 26,171 single nucleotide polymorphism (SNP) markers in a set of 337 flax (*Linum usitatissimum* L.) germplasm, phenotyped in five environments. We evaluated 14 prediction models and several factors affecting predictive ability based on cross-validation schemes. Models yielded significant variation among predictive ability values across traits for the whole marker set. The ridge regression (RR) model covering additive gene action yielded better predictive ability for most of the traits, whereas it was higher for low heritable traits by models capturing epistatic gene action. Marker subsets based on linkage disequilibrium decay distance gave significantly higher predictive abilities to the whole marker set, but for randomly selected markers, it reached a plateau above 3000 markers. Markers having significant association with traits improved predictive abilities compared to the whole marker set when marker selection was made on the whole population instead of the training set indicating a clear overfitting. The correction for population structure did not increase predictive abilities compared to the whole collection. However, stratified sampling by picking representative genotypes from each cluster improved predictive abilities. The indirect predictive ability for a trait was proportionate to its correlation with other traits. These results will help breeders to select the best models, optimum marker set, and suitable genotype set to perform an indirect selection for quantitative traits in this diverse flax germplasm collection.

## Introduction

Flax (*Linum usitatissimum* L.), a natural source of oil and fiber, has been grown throughout the world since prehistoric times and has considerable economic importance^1^. Flaxseed plays an important role in human nutrition^2,3^ by providing oil rich in omega-3 fatty acid, dietary fibers, and anti-carcinogenic lignans. Flaxseed oil is used for different industrial purposes such as ink making, varnishing, painting, and road carpeting due to its specific drying properties^4^. Additionally, flaxseed meal has value as poultry and animal feeds^5,6^ while flax fiber is used in making linen cloth and different bio-industrial products^7^.

Among the flax-growing countries, Kazakhstan produces the most oilseed flax followed by the Russian Federation, Canada, China, and the USA, whereas France alone produces three-fourths of fiber flax in the world followed by Belgium, Belarus, Russian Federation, and China^8^. In the United States, North Dakota (ND) has the greatest % of cultivated flax acres, which covers about 71% (215 million acres) and 80% (3.75 billion bushels) of U.S. flax acreage and production, respectively, and annually contributes about $46 million U.S. dollars to the national economy (Data averaged from 2017 to 2021)^9^. Like many other cultivated crops, flax production in ND is being challenged by different biotic and abiotic stresses^10,11^. To combat these challenges and meet farmers’ desires for high-yielding varieties with increased oil and protein content, North Dakota State University (NDSU) runs a moderate-size flax breeding program. The program utilizes classical breeding methods, especially modified bulk methods to develop varieties, which is expensive, laborious, and time-consuming. To speed up the breeding process, it is prime time to adopt cutting-edge breeding tools such as marker-assisted selection (MAS) and genomic selection (GS) in the program.

Initially, breeders utilized marker-trait associations revealed by linkage mapping and genome-wide association mapping for MAS in breeding programs to enhance efficiency and genetic gain^12^. To date, MAS has been successfully used to improve monogenic or oligogenic traits in many major crops such as rice^13,14^, wheat^15–17^, maize^18–20^, etc. However, the improvement of quantitative traits controlled by multiple QTLs with minor effects is challenging. A multi-marker MAS system can be used to improve quantitative traits, but it is very difficult to identify and account for all the allele effects^21,22^. Breeders can overcome the limitations of MAS by using a genome-wide selection approach. Genomic selection (GS), also known as genomic prediction, by considering all marker effects regardless of significance holds a promise to accelerate the rate of genetic gain in the case of quantitative traits^23,24^. The GS was first successfully used in animal breeding, where it was applied to dairy cattle^25^. In recent days, the low genotypic cost compared to phenotypic cost has made the GS an attractive decision tool to select and evaluate accessions in diverse germplasm collections.

To date, many statistical models for GS have been developed. Initially, the RRBLUP model was widely used^23,26^. Later, plethora of linear or parametric models^27–30^ and non-linear or non-parametric models^31–33^ have evolved. Linear models capture only additive gene action effects, whereas non-linear models capture additive and non-additive (dominance, epistasis, and pleiotropy) interactions. Different models vary in their underlying assumptions and algorithms and many authors confirmed that no single model worked best across traits, rather a particular model outperformed for a particular trait^34–37^. That is why it is always recommended to test multiple models across traits to achieve maximum prediction accuracy. Along with models, various factors such as relatedness between the training set and validation set^38,39^, correlation among studied traits^40,41^, trait heritability, marker density, QTL size with effects^34,42–46^ and genetic diversity of studied collection^36,47,48^ also affect the prediction accuracy.

Plant breeders have successfully utilized GS to accelerate the varietal development process in rice^49–52^, wheat^53–57^, maize^58–61^ and other crops. Despite the wide application of GS in many crops, its utilization in flax breeding has not flourished yet. Because very few reports are available regarding the utilization of GS in bi-parental^62,63^ and diverse^64^ flax populations, there is great potential for evaluating GS in diverse flax collections. This study aims to (1) investigate the feasibility of implementing genomic prediction for various agronomic traits, (2) identify the most effective prediction models and optimal marker numbers to maximize predictive abilities across traits, and (3) assess how marker-trait associations, population structure, and trait correlations influence predictive abilities for diverse traits.

## Materials and methods

### Plant materials

We collected 500 flax accessions and their wild relatives from the North Central Regional Plant Introduction Station (NCRPIS), Ames, Iowa, USA. All genotypes were grown in the field as single rows. We discarded the heterogeneous rows and kept the homogeneous lines for parental stock. Finally, we made a core collection of 337 flax germplasm accessions, which comprises homogeneous lines from NCRPIS, NDSU-released varieties and advanced breeding lines, and varieties developed by different institutes in the USA and Canada (Supplementary Table S1). The advanced breeding lines (F_7_ generation) were obtained by crossing different parents in various combinations. The core collection is being maintained through selfing. NCRPIS and NDSU are public institutions that comply with all required regulations for utilizing seed materials for research and development purposes.

### DNA extraction, sequencing, and SNP calling

Young leaves collected from 30-day old plants were used as the source of DNA. The collected leaf samples from each genotype were lyophilized and subsequently pulverized using stainless beads in a plate shaker. DNA was extracted from the ground leaf tissue using a Qiagen DNeasy Kit (Qiagen, CA, USA) according to the manufacturer’s protocol. A NanoDrop 2000/2000c Spectrophotometer (Thermofisher Scientific) was used to measure the DNA concentrations. The GBS library was prepared using the ApekI enzyme^65^ and sequencing of the library was accomplished using an Illumina HiSeq 2500 sequencer at the University of Texas Southern Medical Center, Dallas, Texas, USA. Identification of SNPs was based on a 120-base kmer length and minimum kmer count of ten using the TASSEL 5 GBSv2 pipeline^66^ and the flax reference genome^67^ (available at: https://ftp.ncbi.nlm.nih.gov/genomes/all/GCA/000/224/295/GCA_000224295.2_ASM22429v2/). The reads were aligned to the reference genome using the Bowtie 2 (version 2.3.0)^68^ alignment tool, which identified 243,040 SNPs that passed all required steps of the TASSEL 5 GBSv2 pipeline. Though flax is a strictly self-pollinating crop and inbred lines were used, there was a possibility of heterozygous SNPs due to artefactual collapse of homologous sites during alignment. We removed the heterozygous SNPs and filtered the row SNP set using VCFtools^69^ following the criteria: minor allele frequency (MAF) ≥ 0.05, missing values (max-missing) ≤ 25%, depth (minDP) ≥ 3, min-alleles = 2 and max-alleles = 2. This filtering process yielded 26,171 bi-allelic high-quality SNP markers.

### Phenotyping

We planted 337 genotypes following an augmented row-column design^70^ with three standard checks (ND Hammond, Gold ND, and Omega), and the checks were diagonally placed to cover spatial heterogeneity (Supplementary Fig. S1). Each check was replicated 20 times per trial and experiments were conducted at Fargo, ND, USA (46.8772° N, 96.7898° W) for three consecutive years (2018, 2019, and 2020) and at Carrington, ND, USA (47.4497° N, 99.1262° W) in two consecutive years (2019, 2020). Hereafter, we referred to the location-year combinations as environments: E1 (Fargo, 2018), E2 (Fargo, 2019), E3 (Carrington, 2019), E4 (Fargo, 2020), and E5 (Carrington, 2020). In E1, we planted a single row (4 m long) per genotype, 7 gm of seeds per row due to a shortage of seeds. Later on, for all environments, for each genotype, we used 8 (4 m × 2 m) m^2^ four-row plots and 30 gm of seeds per plot for planting. Standard fertilization and cultural practices were used throughout the experiment. Data for nine agronomic traits was recorded in all environments and seed yield in four environments. For each genotype, data measured on different traits was based on the criteria and methods described by Nôžková et al. (2011)^71^ with minor modifications. Days to flowering was determined as the number of days from planting to when approximately 50% of plants per plot start flowering. Plant height was measured as the length of the main stem at maturity from the hypocotyl ending point to the plant’s top. The technical length was measured as the length of the main stem at maturity from the end of the hypocotyl to the point where branching starts. The branch number per plant was counted as the lateral branches of the main stem inflorescence. In this case, only the primary lateral branches were considered. The boll number per plant was the capsule number carried by the main stem inflorescence. Thousand seed weight was the weight of exactly 1000 seeds. Seed area, seed width, and seed length were calculated as an average of 1000 seeds. We used a MARVIN seed analyzer (GTA Sensorik GmbH) to measure seed-related traits. We harvested each plot separately and measured the grain weight in grams as yield per plot.

### Phenotypic data analysis

In this study, a two-stage analysis of phenotypic data was performed. In stage I, the best linear unbiased estimates (BLUEs) and other statistics for all genotypes within each environment were determined using the following model:

1 $$y=X\tau +{e}_{R}+{e}_{C}$$

where *X* is the design matrix, *τ* is the fixed effect of genotype, *e*_R_ is the random effect of row and *e*_C_ is the random effect of the column.

In stage II, we fitted the BLUEs and weights from stage I analysis in Eq. (2) and estimated the best linear unbiased predictions (BLUPs) of genotypes across all environments.

2 $${y}_{ij}=\mu +{G}_{i}+{E}_{j}+{GE}_{ij}+{e}_{ij}$$

where ${y}_{ij}$ is the observed phenotypic value of the *i*th genotype in the *j*th environment, *μ* is the overall mean, ${G}_{i}$ is the random effect of the *i*th genotype, ${E}_{j}$ is the fixed effect of the *j*th environment, ${GE}_{ij}$ is the G × E interaction term and ${e}_{ij}$ is the residual error. The analysis was done using the R-shiny app MrBean (https://beanteam.shinyapps.io/MrBean/).

We calculated the heritability of each trait in each environment and combined all environments using the following formula proposed by Cullis et al. (2006)^72^:

3 $${H}_{Cullis}^{2}=1-\left(\frac{PEV}{md*{V}_{g}}\right)$$

where the genotypic predicted error variance is $PEV$, ${V}_{g}$ is the genotypic variance and $md$ is mean values from the diagonal of the relationship matrix. The heritability calculation was done using the R package Sommer^73^.

We calculated Pearson correlation among different traits within each environment and combined all environments using observed unadjusted phenotypic values. The correlation of phenotypic values of a trait observed in different environments was also calculated; for this purpose, we used the R package corrplot^74^.

### Structure and linkage disequilibrium (LD) analysis

An admixture model-based structure analysis of the whole germplasm set was conducted using STRUCTURE^75^ software utilizing the whole SNP marker set (26,171). To strengthen the result, structure analysis was run at various combinations of burn-in lengths (5000–50,000) and Monte Carlo Markov Chain (MCMC) lengths (5000–100,000). Each combination was replicated 10 times per K (K1–K10). We used both the Delta K approach^76^ and four alternative statistics^77^ to identify the optimum number of clusters as the Delta K approach gives a variable number of clusters at different combinations of burn-in lengths and MCMC lengths^120,121^. StructureSelector^78^ was used for this purpose. We assembled 10 replicates of the Q-matrix for the best-fitted cluster number using CLUMPP^79^. Principal component analysis (PCA) was run using a covariance standardized approach in TASSEL ^80^. To show the genetic divergence among identified clusters, pairwise *F*_*st*_^81^ was calculated using Arlequin3.5^82^ at 10,000 permutations. Gaussian finite mixture model-based clustering of the collection was fitted via the EM algorithm in R package mclust^83^ using phenotypic data. We also visualized the PCA and phenotype-based clustering output using the ggplot2 R package^84^.

Chromosome-wise LD (*r*^2^ values) among SNPs was calculated using the 26,171 SNP markers in PopLDdecay software^85^. The LD decay rate was defined as a half-decay distance, at which observed *r*^2^ between sites decays to less than half of the maximum *r*^2^ value^86^. For this purpose, we wrote R scripts combining various R-packages (available on personal communication).

### Genomic prediction models’ comparison

In the case of genomic prediction, the linear model equation is unsolvable as the explanatory variables (marker number) exceed the observation number. Researchers solve this problem by using ridge regression or Bayesian computations or parametric method and penalized regression or semi-parametric method^87^. Generally, all methods are fitted to the basic skeleton (Eq. 4) with modifications:

4 $${y}_{ij}= {\varvec{\upmu}}+Zu+\upvarepsilon$$

where *y* is the phenotypic value (BLUP), µ is the fixed intercept, *Z* is the marker matrix, µ is the marker effect vector and ɛ is a residual vector.

In this study, we assessed the predictive ability of different models for different traits using 26,711 SNP markers and BLUP values. We used 14 different parametric and semi-parametric models such as GBLUP^88–90^, EGBLUP^91^, RR^23,26,92^, LASSO^93^, EN^94^, BRR^95^, BA^23^, BB^96^, BC^97^, BL^29^, RKHS^98^, RF^99^, SVM^100,101^ and MKRKHS^33^. Details of the models are available in previously published research articles^34,95^. For this purpose, we used the R package BWGS pipeline^34^. For each trait, we assessed the predictive ability as Pearson correlation between genomic estimated breeding values (GEBVs) and phenotypic values (BLUPs) of the validation set (VS). For this, a fivefold cross-validation approach was used, i.e., we randomly selected 80% of the collection as a training set (TS) and the remaining 20% as a validation set (VS). The process was repeated 100 times for each model and finally, the predictive ability was reported as average across 100 replicates. One-way ANOVA was done to explore whether the variation among predictive ability values by different models for each trait was significant or not. Then lettering was done by multiple comparison (Tukey) test to separate the averaged predictive ability values into groups. The model that gave the best predictive ability for a particular trait was declared as the best-fitted model for the corresponding trait. For subsequent analyses, we only used the best-fitted model identified in this stage for specific traits.

### Marker subsets preparation

The predictive ability for each trait was assessed using different subsets of the whole marker set. The marker subsets were made based on linkage disequilibrium (LD) decay distance and random selection. We thinned the whole marker set using chromosome-wise LD decay distance, which yielded 5362 markers. For this purpose, we used chromosome-wise half-decay distance, at which observed *r*^2^ between sites decays to less than half of the maximum *r*^2^ value ^86^. We also selected subsets of 20, 200, 1000, 3000, 7000, and 13,000 markers based on random sampling to minimize or avoid any biases. Using a five-fold cross-validation approach, the predictive ability for each subset was measured. The process was repeated 100 times and finally, predictive ability was reported as average across 100 replicates.

### Marker subsets based on marker-trait association

Significant markers for each trait were grouped based on genome-wide association mapping (GWAS) results. This was done in five different ways. In the case of scenario-I, we conducted SNP-based GWAS for all traits within each environment (BLUEs) and combining all environments (BLUPs) using different single locus and multi-locus models such as general linear model (GLM)^102^, mixed linear model (MLM)^103^, compressed MLM (CMLM)^104^, enriched compressed MLM (ECMLM)^105^, settlement of MLM under progressively exclusive relationship (SUPER)^106^, multiple loci MLM (MLMM)^107^, fixed and random model circulating probability unification (FarmCPU)^108^ and Bayesian information and linkage-disequilibrium iteratively nested keyway (BLINK)^109^. The R package GAPIT (version 3)^110^ was used to run GWAS and the best-fitted model for a particular trait was determined based on the mean of squared difference (MSD) values and QQ plots. Using the best-fitted model output, we identified significant SNPs associated with a particular trait based on a *p*-value threshold. The *p*-value threshold was calculated by dividing the type-I error rate (*α*) by the effective number of independent tests (*Meff*) at *α* = 0.05^111^. In this study, the *p*-value threshold was 0.000103. We grouped the significant markers for each trait considering each environment (BLUEs) and combined environment (BLUPs). In the case of scenario-II, GWAS was conducted using only an MLM model based on combined environment BLUP values. The 20 markers for each trait having the lowest *p*-value were grouped. Then the selected marker set from scenario-I & II were used to assess predictive ability 100 times using a five-fold cross-validation approach and predictive ability was reported as average across 100 replicates. In the case of scenario-III, we randomly divided the whole collection into TS (80% of the whole collection) and VS (20% of the whole collection) 15 times. Each time we did GWAS using TS only following the MLM^103^ model and grouped the most significant 20 markers for each trait. Then, predictive ability was assessed and reported as the average across 15 replicates. In the case of scenario-IV, GWAS was conducted as one-way ANOVA using the R function *lm*, and every marker was tested one at a time using phenotype (BLUP values) considering the whole collection. The markers having a *p*-value less than 0.001, 0.01, and 0.05, respectively were grouped separately and then were used for predictive ability calculation 100 times with five-fold cross-validation. In the case of scenario-V, GWAS was done as one-way ANOVA using TS only to select the marker set having a *p*-value less than 0.001, 0.01, and 0.05. The selected marker set was then used to assess the predictive ability for each trait.

### Predictive ability considering population structure

We investigated the confounding effects of population structure on predictive ability. To minimize the effect of population structure, we used genotypic subsets having less divergent genetic clusters and incorporated the Q-matrix from structure analysis output into the RR-BLUP model. Genotypic subsets were made by discarding the most divergent (clusters showing the highest pairwise *F*_*st*_ value to other clusters) genetic clusters P3 and P4 from the whole collection. Albeit a smaller sample size, we also assessed cluster-wise predictive ability. In all cases, we used five-fold cross-validation 100 times.

To explore the effect of population structure, we did stratified sampling to cover maximum genetic variance and calculated predictive ability following two different methods (M-I and M-II). The whole collection was arranged into small groups of 25, 50, 75, 100, 125,150, 175, 200, 250, and 300 genotypes by randomly selecting genotypes from each cluster proportional to the size of the cluster. In the case of method-I (M-I), 100 times five-fold cross-validation within each subset was done to report the predictive ability as the average across all runs. In the case of method -II (M-II), each subset was used as TS and the remaining genotypes as VS. For this purpose, we made 20 replicates of each subset. The predictive ability was calculated as Pearson correlation between GEBVs and BLUP values of VS and was reported as an average across 20 times. There was overlapping of genotypes among replicates as genotypes were selected randomly from each cluster proportional to the size of the cluster each time.

### Indirect predictive ability calculation

Indirect predictive ability for each trait, considering other traits separately, was calculated using the five-fold cross-validation schemes 100 times. For example, we estimated the GEBVs of the validation set for plant height. Then we calculated the indirect predictive ability for seed yield by plant height as Pearson correlation between GEBVs of the validation set based on plant height and BLUP values of that set considering seed yield. The same was repeated 100 times and predictive ability was reported as average across 100 replicates. The same procedure was followed for different trait combinations.

## Results

### Phenotypic variability

The genotype collection showed continuous variation for all traits under all environments (Supplementary Fig. S2). Under the five environmental conditions, days to flowering ranged from 36 to 67, plant height ranged from 20.0 to 82.8 cm, technical length ranged from 6.5 to 60.7 cm, branch number ranged from 2.7 to 12.3 and boll number ranged from 5 to 50. Among the traits, for the boll number, a maximum CV value of 36.2% was observed in E3. However, seed-related traits had relatively lower CV values compared to other traits. Among these, thousand seed weight had a maximum CV value of 16.0% in E1. Seed yield was evaluated under four environments, which ranged from 87 to 630 g per plot with a CV of 35.7 to 41.4%. Boll number and seed yield had the lowest heritability (< 0.40) compared to other traits under all environments. Days to flowering, branch number, and seed width indicated both low to high (0.18 to 0.93) environment-specific heritability, whereas it was high (> 0.60) for plant height, technical length, thousand seed weight, seed area, and seed length. All the traits exhibited high heritability (> 0.65) combining all environments except the branch number. The details of phenotypic variability are presented in Supplementary Table S2.

### Phenotypic correlation

We investigated the phenotypic association among different traits and seed yield (Supplementary Fig. S3) in all environments. Days to flowering showed both positive and negative weak correlations with other traits across environments. Plant height, technical length, branch number, boll number, and seed yield had a positive significant correlation among them across environments. Among all combinations, the best positive significant correlation was found between plant height and technical length (*r* > 0.77), boll number and seed yield (*r* = 0.69 to 0.79) in all environments. Seed-attributing traits such as thousand seed weight, seed area, width, and length were significantly positively correlated with each other, but they exhibited mostly weak correlations with the remaining traits and seed yield across environments.

We also calculated the correlation among environments for each trait (Supplementary Fig. S4) and found comparatively low positive associations among environments for days to flowering and branch number, whereas it was good and positive for the remaining traits.

### Population structure and linkage disequilibrium (LD) analysis

Structure analysis revealed 3 to 9 clusters based on the Delta K approach^76^ and 4–5 clusters based on four alternative statistics (MedMedK, MedMeaK, MaxMedK, and MaxMeaK)^77^ (Table 1). Based on the structure output, we separated the whole collection into five clusters (P1–P5). Winter-type Hungarian (European), spring-type Asian (Indian and Pakistani), and winter-type Turkish genotypes were dominant in clusters P1, P3, and P4, respectively. Cluster P2 contained mixed-type genotypes of different origins while cluster P5 was dominated by spring type NDSU advanced breeding lines of American origin and fiber type (Supplementary Table S1, Supplementary Fig. S5). Here, the type and origin of all genotypes were mentioned according to GRIN-Global database (https://www.grin-global.org/).

**Table 1:** Number of clusters of the genotype collection based on Delta K approach^76^ and four alternative statistics^77^ using different combinations of burn-in lengths and Markov Chain Monte Carlo (MCMC) lengths.

Structure run #	Burn-in lengths	MCMC lengths	Number of clusters (K)	Number of Reps	Number of clusters				
					∆K^α^	Med MedK^β^	Med MeaK^β^	Max MedK^β^	Max MeaK^β^
1	5000	5000	10	10	9	4	4	5	5
2	10,000	10,000	10	10	9	4	4	5	5
3	10,000	50,000	10	10	3	4	4	5	5
4	20,000	20,000	10	10	9	4	4	5	5
5	20,000	50,000	10	10	6	4	4	4	4
6	50,000	50,000	10	10	7	4	4	5	5
7	50,000	100,000	10	10	6	4	4	5	5

^α^The ad hoc ΔK method. ^β^ the median (MedMedK and MaxMedK) or mean (MedMeaK and MaxMeaK) estimators to determine the number of cluster (K).

**Figure 1:**
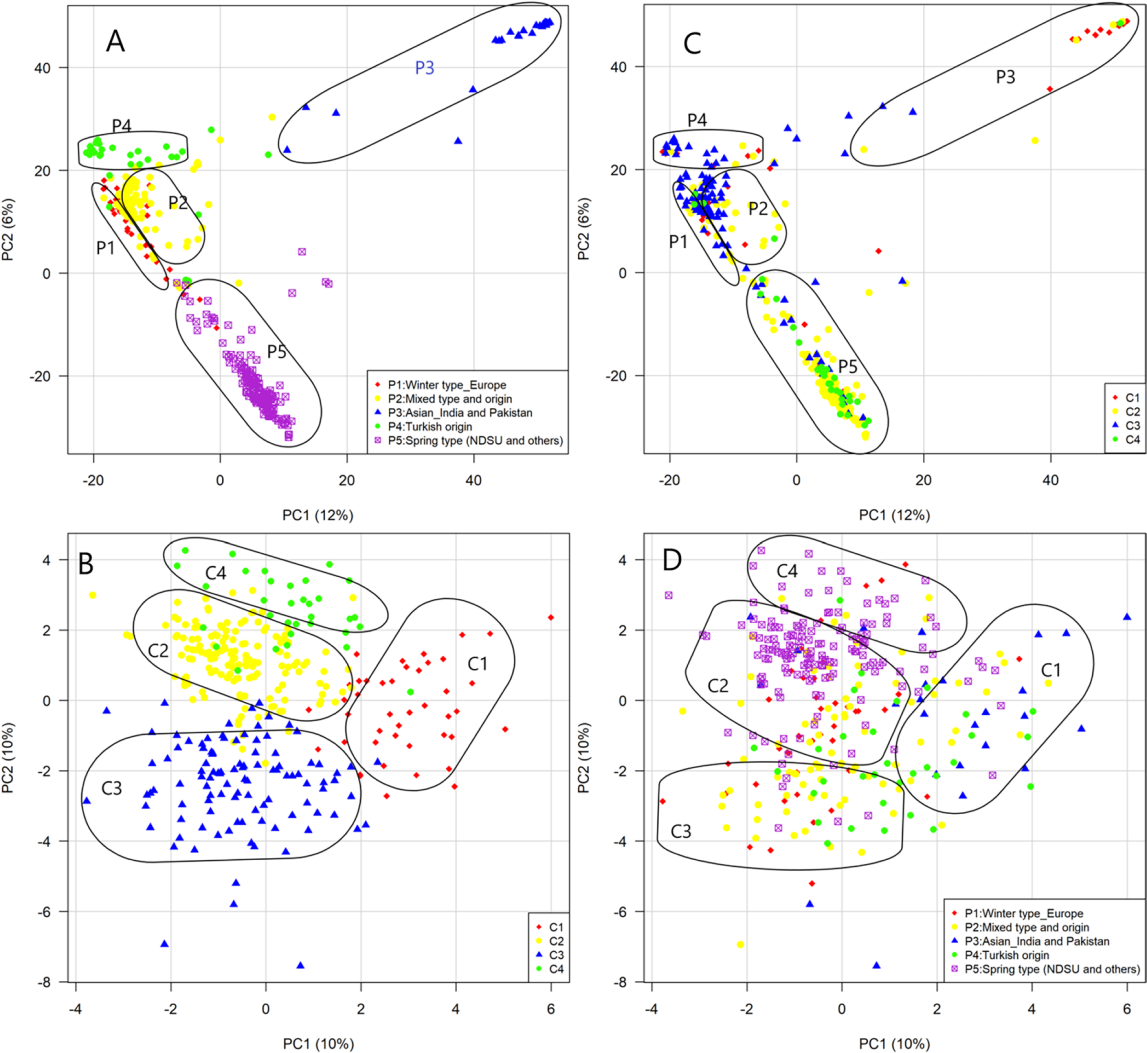
Genotype (SNP markers) and phenotype-based clustering of the whole collection. (**A**) Principal component analysis of SNP diversity based on genetic distance. Colors represent clusters identified at K = 5 in Supplementary Fig. S5. (**B**) Principal component analysis of the whole collection using phenotypic data. Colors represent groups identified by the Gaussian finite mixture model. (**C**) Genotypic clusters showing genotypes belong to different phenotypic groups. (**D**) Phenotypic groups showing genotypes belong to different genotypic clusters.

We also performed principal component analysis (PCA) to show the genetic similarity among genotypes. The first two axes explained 18% of the total observed variation. The output of principal component analysis was in line with that of structure analysis (Fig. 1A). A pairwise *F*_*st*_ comparison indicated genetic divergence among clusters. All combinations showed significant pairwise *F*_*st*_ comparison at *p* < 0.01. We found *F*_*st*_ ≥ 0.20 for all combinations except combinations P1 and P2, P2 and P4. Cluster P3 showed maximum divergence (*F*_*st*_ ≥ 0.30) from all other clusters (Table 2).

**Table 2:** Genetic differentiation among different clusters.

	Cluster pairwise *F*_*st*_				
	P1	P2	P3	P4	P5
P1	0				
P2	0.13**	0			
P3	0.48**	0.38**	0		
P4	0.21**	0.13**	0.47**	0	
P5	0.24**	0.20**	0.50**	0.30**	0

Diagonal values are pairwise *F*_*st*_ values based on 10,000 permutations using Arlequin v. 3.5. **indicates *p* ˂ 0.01.

Moreover, Gaussian finite mixture model-based clustering of the whole collection using phenotypic data yielded four clusters (C1 to C4) (Fig. 1B). Cluster C1, C3, and C4 contained genotypes of different types and origins, while cluster C2 contained the highest number of genotypes dominated by spring type NDSU advanced breeding lines (Supplementary Table S1). Phenotype-based clustering was not consistent with genotype-based clustering (Fig. 1C, D) i.e., genotypic clusters containing genotypes belong to different phenotypic clusters and vice-versa.

In the whole collection, LD decayed to its half maximum within < 21 kb. LD decay rate varied according to chromosome (Supplementary Fig. S6), which was slowest in chromosomes Lu1 and Lu3 (32 kb) but was fastest in chromosomes Lu7 and Lu8 (15 kb).

### The efficiency of different genomic prediction models

**Figure 2:**
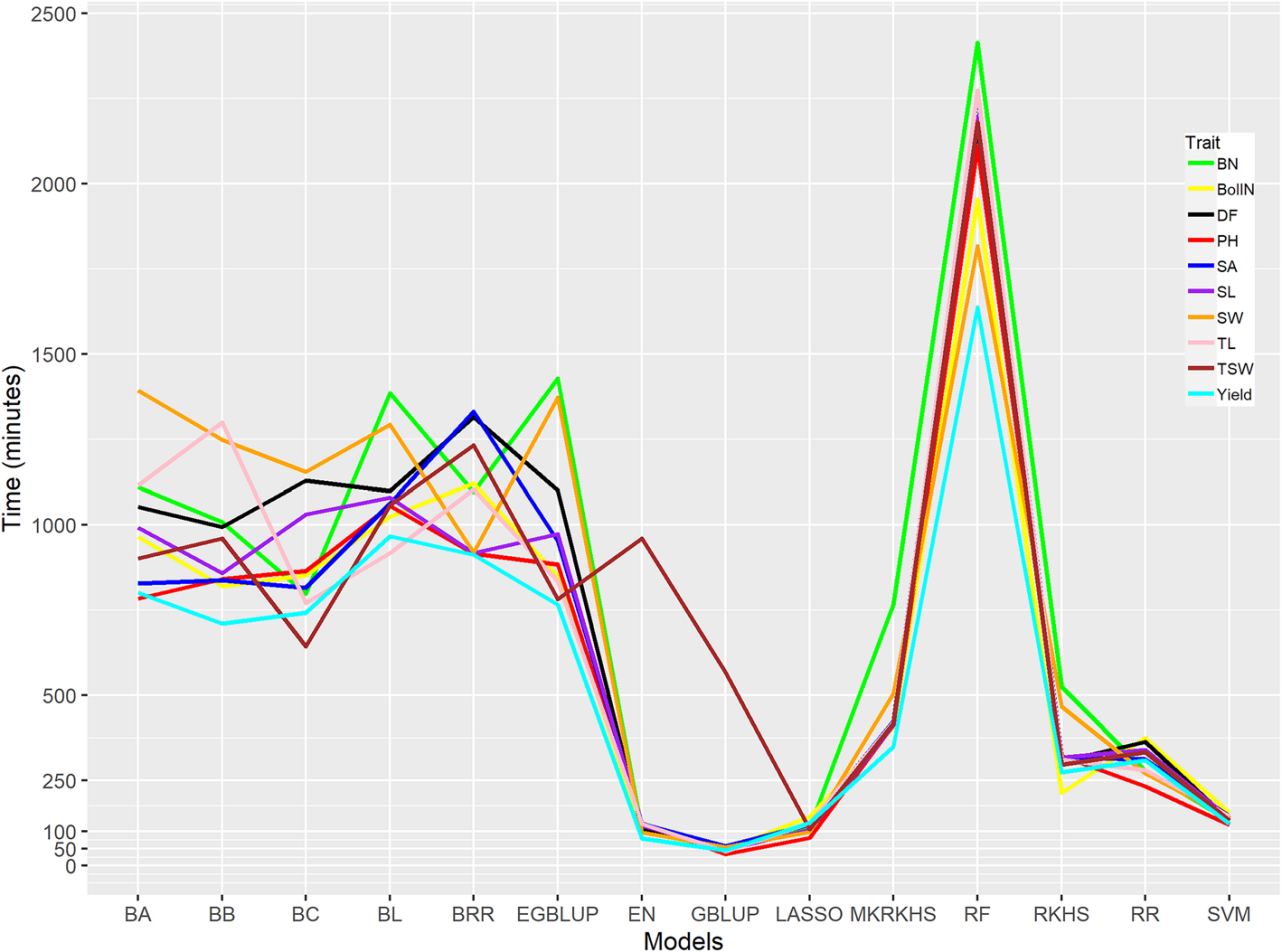
Line graph showing computing time required for running different models for different traits. Each model was replicated 100 times. DF is days to flowering, PH is plant height, TL is technical length, BN is branch number, BollN is boll number, TSW is thousand seed weight, SA is seed area, SW is seed width and SL is seed length.

**Figure 3:**
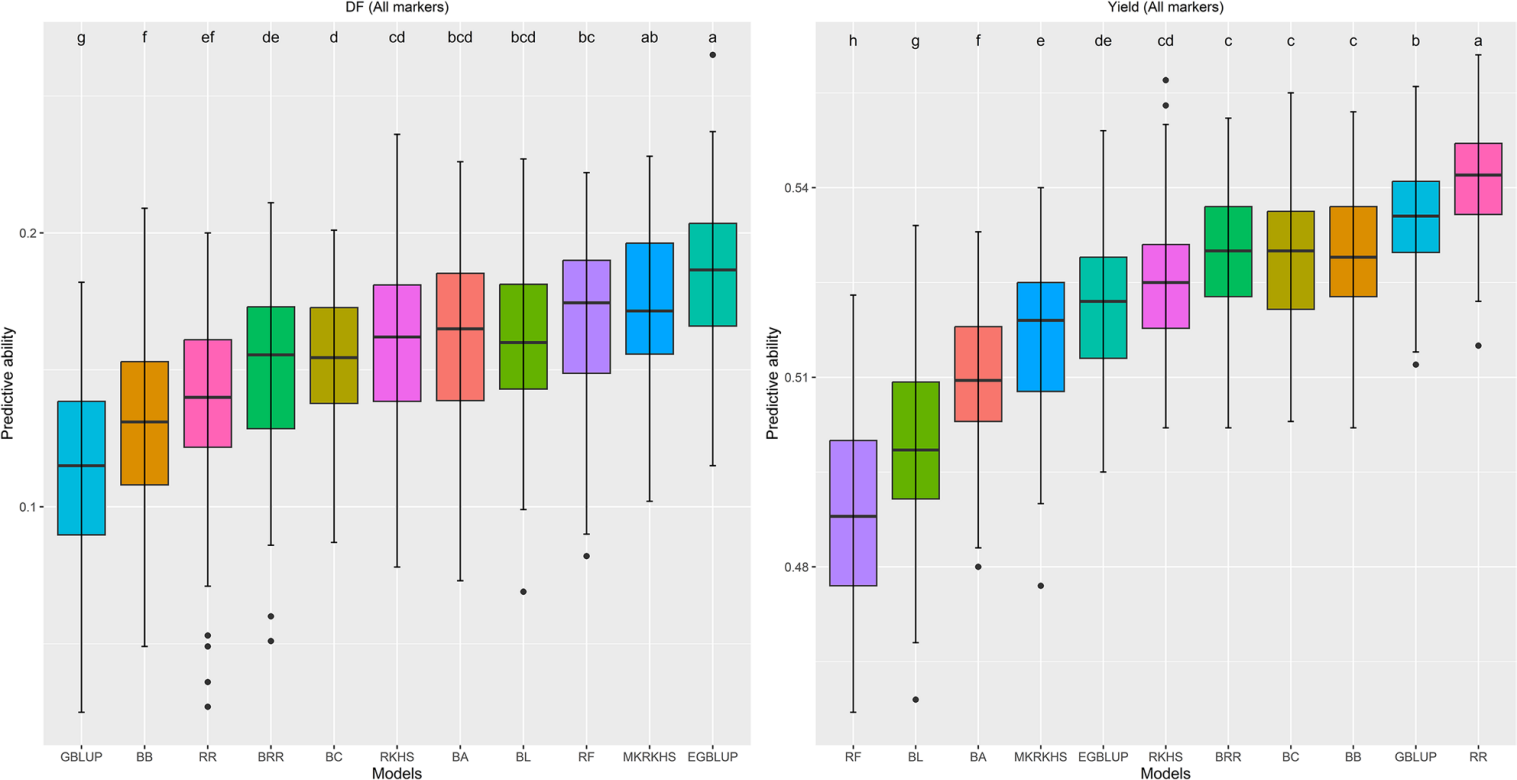
Boxplot showing the distribution of 100 replicates of predictive ability for days to flowering (DF) and seed yield using 14 models. SVM, LASSO, and EN model was not shown due to their low predictive ability. Boxplot for all traits were presented in Supplementary Fig. S7.

We determined the efficiency of 14 genomic prediction models in terms of computing time requirement and predictive ability (Figs. 2 and 3). For all traits, GBLUP required less time (< 60 min.) except the trait thousand seed weight (567 min.) and RF was the most time-demanding (1637–2414 min.) model. EGBLUP, a modification of the GBLUP model, which covers epistatic interactions, required more time (767–1428 min.) than GBLUP. Besides GBLUP, other less time-demanding models were EN (51–124 min.), LASSO (82–143 min), SVM (120–155 min.), and RR (233–374 min.). As time requirement by different models is heavily affected by computer configurations, we always used the same set-up for each model and the values presented here must be taken only for comparison.

The predictive ability values by different models vary significantly (*p*-value < 0.001) for each trait (Fig. 3, Supplementary Fig. S7). For all traits, SVM yielded the poorest predictive ability (− 0.03 to 0.09). LASSO and EN also resulted in low predictive ability for all traits. Model RR yielded the highest predictive ability for plant height (0.48), technical length (0.56), boll number (0.54), thousand seed weight (0.48), seed width (0.49), and seed yield (0.54). EGBLUP had the best predictive ability for days to flowering (0.19), and branch number (0.20), and RF had the best predictive ability for seed area (0.56), and seed length (0.56). The standard GBLUP model did not yield the highest predictive ability values for any traits and its magnitude was significantly (*p*-value < 0.001) lower compared to various models (Fig. 3 and Supplementary Fig. S7).

### Predictive ability considering various marker subsets

We found significant (*p* < 0.001) variation among the predictive ability values for each trait according to various marker subsets (Table 3 and Supplementary Table S4). For all traits, predictive ability was lowest for 20 randomly selected markers, and it increased with the increment of marker numbers. Randomly selected 13,000 markers yielded the highest predictive ability for traits days to flowering, thousand seeds weight, seed length, and yield, whereas it was highest by 7000 randomly selected markers for traits plant height, technical length, and seed area. Marker subset based on linkage disequilibrium decay showed the highest predictive ability for remaining traits. Utilization of the whole marker set did not yield the highest predictive ability values for any traits.

**Table 3:** Predictive ability based on randomly selected markers for different traits.

Marker selection criteria	No. of markers	Predictive ability									
		DF	PH	TL	BN	BollN	TSW	SA	SW	SL	Yield
All marker	26,171	0.19	0.48	0.56	0.20	0.54	0.48	0.56	0.49	0.56	0.54
All marker*	26,171	0.23	0.52	0.60	0.38	0.63	0.50	0.57	0.53	0.59	0.58
LD pruning	5362	0.15	0.48	0.56	0.21	0.54	0.48	0.55	0.49	0.54	0.54
RS	13,000	0.19	0.48	0.56	0.20	0.54	0.49	0.55	0.49	0.55	0.54
RS	7000	0.18	0.48	0.56	0.20	0.54	0.48	0.56	0.48	0.54	0.54
RS	3000	0.17	0.48	0.55	0.19	0.54	0.47	0.55	0.48	0.55	0.54
RS	1000	0.14	0.49	0.56	0.20	0.54	0.48	0.54	0.48	0.54	0.54
RS	200	0.14	0.45	0.54	0.15	0.51	0.45	0.52	0.46	0.52	0.50
RS	20	0.04	0.42	0.42	0.06	0.38	0.34	0.42	0.31	0.27	0.33

In all cases, we assessed predictive ability using the best model identified in Fig. 3 and Supplementary Fig. S7. LD pruning = markers were selected based on chromosome-wise LD decay distance, RS = markers were randomly selected. DF is days to flowering, PH is plant height, TL is technical length, BN is branch number, BollN is boll number, TSW is thousand seed weight, SA is seed area, SW is seed width and SL is seed length. For each trait, predictive ability values by different marker subset varies significantly (*p* < 0.001), which was shown in detail in Supplementary Table S4.

* indicate prediction accuracy calculated according to formula proposed by Ould Estaghvirou et al. (2013)^112^.

Marker subsets based on marker-trait associations (scenario-I to V) significantly (*p*- value < 0.001) affect the predictive ability values for all traits (Table 4 and Supplementary Table S5). Scenario- III yielded the lowest predictive ability for all traits, whereas it was highest by scenario-II for traits plant height (0.60), branch number (0.45), boll number (0.61), seed width (0.60), seed length (0.67) and yield (0.64), and by scenario- I for traits technical length (0.63), thousand seeds weight (0.72) and seed area (0.61). Scenario-IV yielded the highest (0.49) predictive ability for days to flowering at *p*-value ≤ 0.01. The predictive ability computed using all markers was better than that of scenario-III but was lower than that of scenario-I & II for all traits (Table 4). At all *p*-levels, the predictive ability for scenario-IV was better than that of scenario-V for days to flowering, technical length, branch number, and thousand seed weight, but opposite results were found for plant height, boll number, seed area, seed width, seed length, and seed yield. The marker number used for predictive ability computation varied according to traits and selection scenarios (Supplementary Table S3).

**Table 4:** Predictive ability based on markers, selected using marker-trait associations following scenario-I, II, III, IV, and V.

Marker selection criteria	*p*-value	Predictive ability									
		DF	PH	TL	BN	BollN	TSW	SA	SW	SL	Yield
All marker	–	0.19	0.48	0.56	0.20	0.54	0.48	0.56	0.49	0.56	0.54
Scenario- I	0.000103	0.28	0.58	0.63	0.28	0.58	0.72	0.61	0.52	0.67	0.61
Scenario-II*	–	0.47	0.60	0.61	0.45	0.61	0.64	0.51	0.60	0.67	0.64
Scenario-III*	–	0.02	0.27	0.17	0.14	0.39	0.33	0.41	0.33	0.40	0.27
Scenario-IV	0.001	0.45	0.49	0.55	0.45	0.53	0.51	0.46	0.52	0.47	0.53
Scenario-V	0.001	0.05	0.51	0.52	0.15	0.56	0.46	0.53	0.51	0.51	0.58
Scenario-IV	0.01	0.49	0.48	0.54	0.43	0.52	0.49	0.44	0.49	0.45	0.52
Scenario-V	0.01	0.11	0.51	0.53	0.11	0.56	0.46	0.53	0.51	0.51	0.58
Scenario-IV	0.05	0.48	0.47	0.53	0.44	0.51	0.48	0.43	0.48	0.43	0.51
Scenario-V	0.05	0.14	0.50	0.53	0.12	0.55	0.46	0.53	0.51	0.51	0.58

Each scenario was discussed in detail in the method section. DF is days to flowering, PH is plant height, TL is technical length, BN is branch number, BollN is boll number, TSW is thousand seed weight, SA is seed area, SW is seed width and SL is seed length. In all cases, we assessed predictive ability using the best model identified in Fig. 3 and Supplementary Fig. S7. For each trait, predictive ability values by different marker subset varies significantly (*p* < 0.001), which was shown in detail in Supplementary Table S5. The number of markers used for different scenarios were mentioned in Supplementary Table S3.

* In the case of scenario-II & III, the *p*-value was not mentioned as it varies according to traits.

### Predictive ability considering population structure

We partitioned the whole collection into five (P1–P5) (Fig. 1A) and four (C1–C4) (Fig. 1B) clusters based on marker genotype and phenotypic data, respectively. To increase the level of relatedness among genotypes, genotypes belonging to clusters P3 and (P3 + P4) were discarded from the whole collection as these two clusters showed the greatest divergence from other clusters and incorporated Q-matrix in the model. In all cases, predictive ability was lower than that resulting from using the whole collection for all traits (Table 5). Predictive ability within each cluster was always lower than that of using the whole collection for all traits. The magnitude of genotype number within each cluster was not reflected by the magnitude of predictive ability i.e., clusters having more genotypes exhibited both high and low predictive ability for different traits and vice-versa (Table 5).

**Table 5:** Comparison of predictive ability based on population structure.

Clusters	Genotype number	Predictive ability									
		DF	PH	TL	BN	BollN	TSW	SA	SW	SL	Yield
Whole set (WS)	337	0.19	0.48	0.56	0.20	0.54	0.48	0.56	0.49	0.56	0.54
WS-P3^a^	312	0.19	0.42	0.52	0.17	0.52	0.49	0.55	0.46	0.55	0.52
WS-(P3 + P4)^b^	280	0.18	0.33	0.40	0.18	0.43	0.49	0.52	0.45	0.53	0.42
SP^+^	337	0.17	0.36	0.34	0.07	− 0.10	0.40	0.41	0.36	0.45	− 0.03
P1	41	0.01	− 0.32	− 0.37	0.33	− 0.16	0.48	0.42	0.35	0.38	− 0.15
P2	83	0.07	− 0.01	− 0.01	0.09	− 0.04	0.04	0.08	0.06	0.09	0.17
P3	25	− 0.27	− 0.44	− 0.47	− 0.25	− 0.44	0.34	0.49	0.46	0.30	− 0.43
P4	32	0.07	− 0.28	− 0.36	− 0.19	0.22	0.12	0.11	0.18	− 0.02	0.0002
P5	156	0.22	0.11	0.14	0.11	0.32	0.22	0.13	0.09	0.18	0.19
C1	43	− 0.29	− 0.25	− 0.09	0.10	− 0.28	− 0.23	0.10	− 0.12	0.06	0.04
C2	156	0.18	− 0.001	0.20	0.14	0.25	0.08	0.15	0.09	0.15	0.14
C3	105	0.20	0.22	0.28	0.13	0.25	0.31	0.19	0.06	0.07	0.21
C4	33	0.09	0.27	0.20	− 0.29	− 0.26	0.25	0.05	0.03	0.14	− 0.25

In all cases, we assessed predictive ability using the best model identified in Fig. 3 and Supplementary Fig. S7 using all markers. ^a^ Genotypes belonging to cluster P3 were discarded for analysis. ^b^ Genotypes belonging to clusters P3 and P4 were discarded for analysis. SP^+^ refers to the population structure addressed in the model. P denotes the cluster identified by structure analysis using SNP marker data in Fig. 1A. C denotes the cluster identified by the Gaussian finite mixture model based on phenotypic data in Fig. 1B. DF is days to flowering, PH is plant height, TL is technical length, BN is branch number, BollN is boll number, TSW is thousand seed weight, SA is seed area, SW is seed width and SL is seed length.

**Figure 4:**
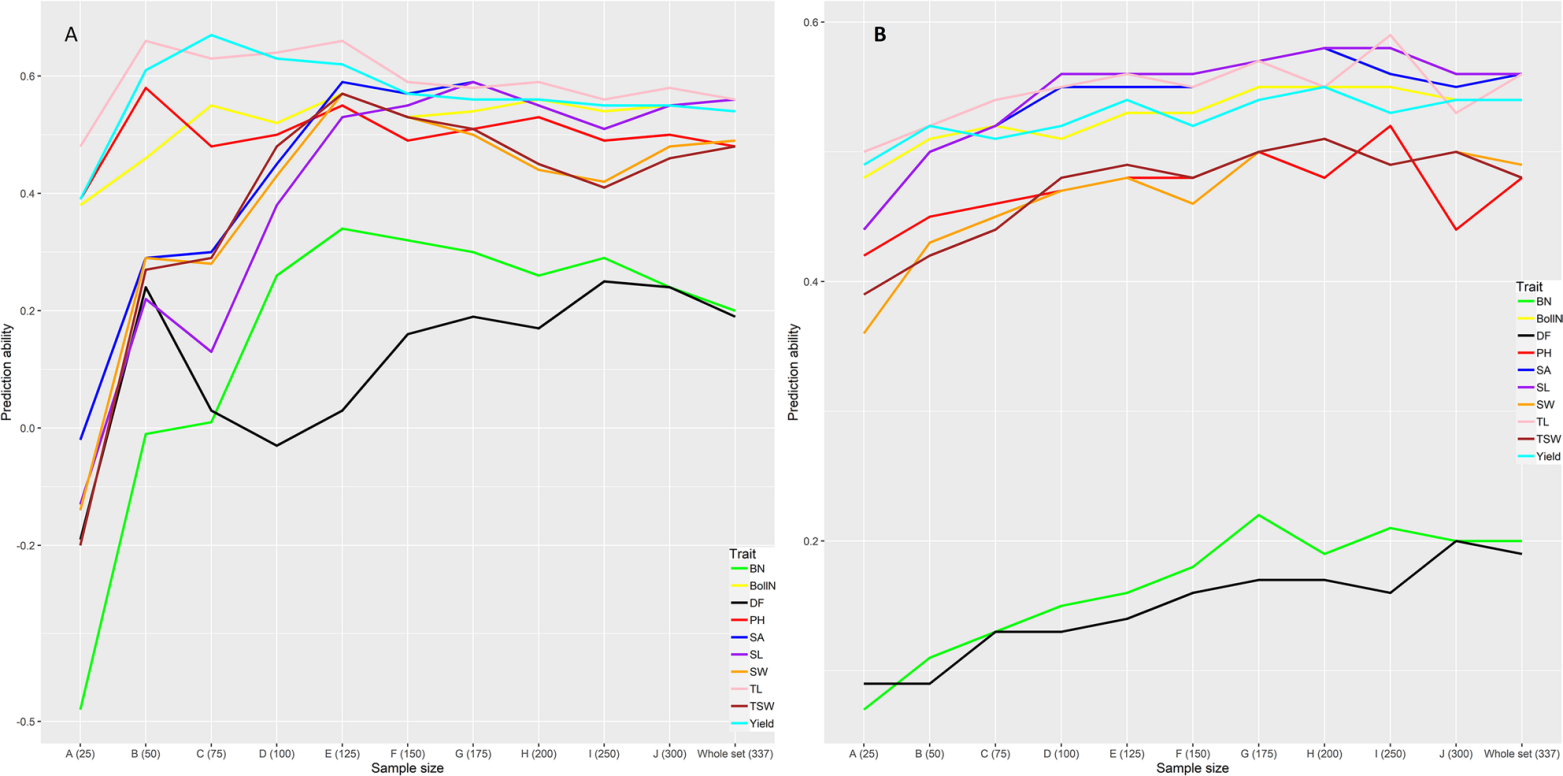
Stratified sampling effects on predictive ability. (**A**) Sampling and prediction ability were calculated according to M-I. Each sample was a training set, and the remaining genotype was a validation set. (**B**) Sampling and prediction ability were calculated according to M-II. Five-fold cross-validation was done within each sample. DF is days to flowering, PH is plant height, TL is technical length, BN is branch number, BollN is boll number, TSW is thousand seed weight, SA is seed area, SW is seed width and SL is seed length.

Stratified sampling and predictive ability assessment were done for all traits according to M-I and M-II. In the case of M-I, the predictive ability based on different sample sizes was better than that of the whole set (Fig. 4A). Sample size 125 yielded the best predictive ability for branch number, boll number, thousand seed weight, seed width, and seed length. For days to flowering, plant height, and technical length, we found the best predictive ability at sample size 50, whereas it was better for seed length and seed yield at sample size 175 and 75, respectively. Likewise, M-I, as in the case of M-II, predictive ability was better based on different sample sizes than that for the whole set across traits (Fig. 4B). For days to flowering, plant height, technical length, branch number, and boll number the predictive ability reached a plateau at sample size 175, whereas, for thousand seed weight, seed area, seed width, seed length, and seed yield the same happened at sample size 200. In most cases, predictive ability based on M-I was better than that based on M-II.

### Indirect predictive ability

The predictive ability of any target trait was calculated using the GEBVs of another trait (Table 6). Trait combinations with the best positive correlation resulted in better predictive ability, except for the combinations of days to flowering and plant height, days to flowering and branch number, and plant height and branch number. For instance, the predictive ability for seed yield based on GEBVs of plant height, technical length, and boll number was 0.46, 0.50, and 0.55, respectively, as seed yield showed a better correlation to plant height (0.25), technical length (0.38), and boll number (0.73). We found similar results for other traits also.

**Table 6:** Indirect predictive ability values for traits considering correlated traits.

Traits	DF	PH	TL	BN	BollN	TSW	SA	SW	SL	Yield
DF	0.19	− 0.16	− 0.16	− 0.09	− 0.01	− 0.05	− 0.07	− 0.08	− 0.02	0.00
PH	0.36	0.48	0.55	0.11	0.43	− 0.20	− 0.27	− 0.15	− 0.30	0.46
TL	0.07	0.78	0.56	0.10	0.48	− 0.21	− 0.30	− 0.17	− 0.33	0.50
BN	0.46	0.69	0.37	0.20	0.15	0.21	0.21	0.26	0.17	0.15
BollN	− 0.01	0.27	0.31	0.14	0.54	− 0.19	− 0.31	− 0.18	− 0.32	0.55
TSW	− 0.04	− 0.21	− 0.21	− 0.12	− 0.04	0.48	0.55	0.48	0.55	− 0.22
SA	− 0.01	− 0.18	− − 0.21	− 0.05	− 0.17	0.3	0.56	0.49	0.57	− 0.27
SW	− 0.05	− 0.16	− 0.10	− 0.10	− 0.06	0.70	0.84	0.49	0.53	− 0.20
SL	0.02	− 0.24	− 0.22	− 0.12	− 0.14	0.74	0.91	0.82	0.56	− 0.29
Yield	− 0.01	0.25	0.38	0.06	0.73	− 0.07	− 0.25	− 0.12	− 0.20	0.54

Diagonal values are direct predictive ability for traits. Above diagonal values are indirect predictive ability values for traits considering the GEBVs of correlated traits shown in column. Below diagonals are correlation coefficient values among traits. In all cases, we assessed predictive ability using the best model identified in Fig. 3 and Supplementary Fig. S7 using all markers. DF is days to flowering, PH is plant height, TL is technical length, BN is branch number, BollN is boll number, TSW is thousand seed weight, SA is seed area, SW is seed width and SL is seed length.

## Discussion

### Phenotypic variability

Here, we investigated seed yield and nine other agronomic traits such as days to flowering, plant height, technical length, branch number, boll number, thousand seed weight, seed area, seed width, and seed length. These traits play an important role in flax development, adaptation, domestication, and improvement^113^. All these traits had continuous variation in all environments suggesting polygenic inheritance. Correlations among polygenic traits occur due to linkage and/or pleiotropic effect^114^. The better the correlations among traits the more likely it is that breeders can indirectly select one trait based on other traits with high heritability. In the current study, we found a very good positive correlation between plant height and technical length, and among seed-related traits. This finding will allow breeders to phenotype only plant height or technical length and any seed-related traits for further research using this germplasm collection, which will greatly reduce the phenotyping and analysis workload. Among the traits, boll number had the best correlation with seed yield. Previous studies^115,116^ also confirmed the most direct contribution of boll or fruit number to flax seed yield. Days to flowering always had a negative correlation to seed yield and seed-related traits, which leads to the assumption that there is a possibility of exhausting more photosynthetic carbohydrates by late flowering flax genotypes for vegetative growth rather than seed formation and development. Seed traits such as thousand seed weight, seed area, seed width, and seed length were also negatively correlated to seed yield, which is consistent with previous studies^117–119^.

### Population structure

Structural variation and phenotypic diversity in a collection are inevitable when a breeder deals with a germplasm collection of different origins, types, and sources. Structural variation occurs due to allelic diversity present in the collection, whereas phenotypic variation is linked to this allelic diversity as well as environmental variations. Structure presence in a population influences its conservation and utilization and affects the output of genome-wide association analysis and genomic prediction. Population structure is influenced by mating strategy, mutation, selection, and gene flow^122^. The clear-cut separation of Asian (P3) and Turkish (P4) genotypes from others indicates that the geographic distance accelerates genetic differentiation by hindering gene flow. The presence of variable types of genotypes having mixed origin in sub-population P2 supports the hypothesis of active exchange of germplasm among European countries^123^ as well as among other countries. The grouping of all NDSU-released varieties and advanced breeding lines and Canadian genotypes under the same sub-population P5 was due to shared ancestors and exchange of germplasm between the USA and Canada^124^. The fiber-type genotypes were in this sub-population as they were part of the parental set used for developing advanced breeding lines. We also partitioned the germplasm collection into four groups using ten quantitative agronomic traits. However, no clear-cut phenotypic clustering pattern according to types and origins was observed; the spring-type NDSU advanced breeding lines dominate one group, whereas spring-types of other origins and fiber type cluster together in another group. The pattern of the genotypic clusters was not reflected by phenotypic grouping and vice versa (Fig. 1). The mismatch between genotypic and phenotypic clustering output was also reported in flax^125^, winged yam^126^, and durum wheat^127^. This mismatch could be improved by incorporating more plant features i.e., traits (qualitative and quantitative) and diverse environments in further studies.

### Genomic prediction

The studied germplasm collection showing considerable genetic diversity will resist genetic erosion, boost genetic gain, and serve as a source of valuable genes for further improvement. As the studied traits had continuous variations, relying on phenotypic evaluation alone can be expensive, laborious, and time-intensive. However, the low genotyping cost, relatively accurate genotyping, and efficient computational algorithms increase the opportunities to evaluate and utilize this collection for genomic prediction, which will reduce the time and cost associated with traits evaluation^128–132^.

### Comparing genomic prediction models

Various genomic prediction models are available, which can capture both linear (additive) and non-linear (epistasis and dominance) effects. In this study, we used predictive ability and time requirement to compare 14 different models. Breeders can also use prediction accuracy to compare the models, which can be calculated by dividing the predictive ability values by the square root of the corresponding traits’ heritability^112^. Although prediction accuracy across all traits was better than corresponding predictive ability values (Table 3), we chose predictive ability as criteria to compare models, since there are possibilities of estimating and interpreting heritability poorly^133,134^.

We found predictive ability values per trait by different models varied significantly, which was opposite^36,42^ as well as similar^35,51,137,138^ to previous reports. For example, Bari et al. (2021)^36^ determined the predictive ability for six traits in a diverse pea germplasm collection using five different models. They observed almost the same predictive ability values across traits by different models. On the other hand, Azodi et al. (2019)^35^, investigated 12 linear and non-linear models for different traits in six species and concluded that predictive ability by different models varies significantly for all traits. Yu et al. (2022)^51^, Phumichai et al. (2022)^137^, and Roorkiwal et al. (2016)^138^ revealed the same phenomenon in rice, cassava, and chickpea, respectively. These reports and our findings confirmed that no single model worked best for all traits i.e., specific models are good for specific traits. Among all models, RR yielded the highest predictive ability for most of the traits, whereas it was lowest by SVM for all traits. A similar performance of SVM was found in wheat^34,135^, but the opposite scenario in maize^136^. The differences in predictive ability values by models per trait and among traits were because of variation in the underlying algorithm of models and the unique complex biology shaping the traits^129,139^. Gene action (additive, dominance, and epistasis) affects prediction accuracy for traits^141,142^. Empirical and theoretical evidence indicates that the lion share of genetic variance is additive, though gene action is not^143,144^. Momen et al. (2018)^37^ reported that linear or parametric, and non-linear or non-parametric models outperform for traits under additive and non-additive gene action, respectively. In our study, for most of the traits model, RR resulted in the highest predictive ability values, which conferred that these traits were under additive gene action. Apart from this, linear model EGBLUP and non-linear model RF yielded the best predictive ability for days to flowering, branch number, seed area, and seed length. Epistatic gene action may shape these traits as outperforming models capture epistatic gene interaction^37,91^. In our study, the predictive ability values of different traits were proportionate to traits’ heritability, indicating heritability affects genomic prediction, which was confirmed by previous reports in many crops^42,43,140^. In our study, branch number having low heritability showed poor predictive ability, whereas both were higher for other traits except days to flowering. In the case of days to flowering, though having high heritability, it showed low predictive ability. This may happen as there is a possibility of estimating heritability poorly^133,134^. Compared to our findings, Lan et al. (2020)^62^ found better predictive ability for days to maturity, but lower predictive ability for seed yield in a bi-parental flax population of 260 lines. The lower predictive ability for seed yield was also supported by You et al. (2016) ^63^ in three different bi-parental flax populations. Overall findings indicate that the breeder should test various models for different traits to select the best-fitted model.

### Marker density effect on predictive ability

Cost-effective next-generation sequencing techniques and the availability of high-quality reference genome have enabled breeders to extract informative genetic markers in prolific numbers. Utilizing these resources and high-performance computing facilities, breeders can feed the models with a huge number of markers. Although many models have been developed to handle the over-parameterization problem (marker number >  > observation number) in genomic selection, previous reports confirmed that adding more markers after a certain number did not improve predictive ability. For example, in a wheat panel of 760 lines, predictive ability reached a plateau above around 5000 randomly selected markers^34^, whereas in maize natural and bi-parental populations it requires 7000 and 2000 randomly selected markers, respectively^141^. We found the same trend of predictive ability in this study where a plateau was obtained around 1000–3000 randomly selected markers. In a recent simulation study, Chang et al. (2018)^44^ achieved the same prediction accuracy by using 0.5 to 1% of all markers compared to that of the whole marker set (200,000). In our case, it happened at around 26% (7000) of total markers. This finding indicates that the predictive ability by marker subset capturing all QTL information and by the whole marker set will be the same. Our finding confirmed this, where predictive ability by a marker subset (5362) based on LD decay distance was higher than that by the whole marker set. In this study, more markers were required to obtain maximum predictive ability, though it required only 256 markers in a wheat bi-parental population^145^, and 1000–1200 markers in a soybean varietal collection of 235 individuals^146^. This discrepancy in marker numbers among various research was due to the nature of the studied population and LD decay pattern. Although fewer markers can capture all QTL information in a bi-parental and varietal collection due to slow LD decay, more markers were required in this germplasm collection due to rapid LD decay.

### Marker-trait association effect on predictive ability

In this study, the GWAS-derived significant SNP subset yielded better predictive ability values than that by the randomly selected marker subset and, surprisingly, even better predictive ability than that by the whole marker set across all traits. This finding was in line with results obtained by other authors in flax^62^, wheat^34,45^, maize^46,147^, and spinach^148^. This overestimation only prevailed when marker selection was made on the whole population (training set + validation set), instead of the training set only, indicating a clear overfitting. That is why in practice, utilization of the marker set yielded by GWAS considering the whole collection is not recommended. This overfitting did not occur when marker selection was done based on a one-way ANOVA approach either using the whole collection or training set across all traits, except for days to flowering and branch number. This was due to capturing more markers (≥ 2000) by the one-way ANOVA approach, as more markers dissolve overfitting by acting like random selection. For future studies, breeders can use marker subsets based on LD decay distance rather than random selection and GWAS-based selection, which will ensure maximum predictive ability and will reduce run time.

### Population structural effect on predictive ability

Because the availability of diverse genetic materials in a breeding program ensures its sustainability, breeders strive to improve predictive ability while maintaining genetic diversity. The studied genotypic collection has substantial genotypic and phenotypic diversity (Fig. 1). We corrected structural variation by discarding most divergent clusters and incorporating the population Q-matrix during analysis. In both cases, predictive ability did not improve across traits. Similar phenomena were found in wheat^149,150^, pea^36,151^, barley^152^ and maize^47,153^. This finding confirms that for quantitative traits, reducing genetic structural variation increases genetic homogeneity in the collection, but not phenotypic homogeneity. We also observed very low to negative predictive ability across traits within each genotypic and phenotypic cluster, though Haile et al. (2021)^154^ found moderate to high prediction accuracies within wheat subpopulations. The reason behind this was the inconsistency between genotypic and phenotypic variance i.e., genotypes of specific genetic clusters were grouped under different phenotypic clusters and the same happened to the genotypes of specific phenotypic clusters (Fig. 1C, D). Smaller population sizes in some clusters may also contribute to this.

In this study, the reduction of predictive ability due to the correction of population structure has driven us to follow a stratified sampling approach, as previous research indicates that stratified sampling^48^ or composite sampling^155^ increases predictive ability. The same has happened in our research as stratified sampling (M-I and M-II) yielded better predictive ability compared to the whole collection. For all traits, in the case of M-I, a small sample size (50–125) yielded better predictive ability compared to the whole collection, whereas it gradually increased with the increment of training size in the case of M-II. In terms of predictive ability, M-I was more productive than M-II, which confirmed that small-sized stratified sample is strong enough to capture diversity as well as maintain good predictive ability in a diverse germplasm collection. In addition, breeders can use genotypes as parents from a particular sample yielding the higher predictive ability to make Multi-parent Advanced Generation Intercrosses (MAGIC) populations for future studies because the relatedness among individuals of the training set and target set accelerates predictive ability^38,39,156^.

### Indirect genomic prediction

In this study, traits had both positive and negative correlations with each other. Breeders can utilize information on correlated traits to predict the target trait using a multi-trait genomic prediction approach. Many previous studies exhibited benefits^40,41,157,158^ and no benefit^159–161^ of multi-trait over single-trait prediction approach in different crops. However, one of the major limitations of the multi-trait approach is that breeders need to phenotype multiple correlated traits, which is expensive and laborious. To overcome this limitation, breeders can use an indirect genomic prediction approach i.e., prediction of genotype for target trait by using a correlated single trait. The benefit of the indirect approach accelerates if the correlated trait possesses high heritability and is easy to phenotype at the crop’s early stages of development and vice-versa for the focal trait. Our findings revealed the benefit of indirect genomic selection as indirect predictive ability based on highly correlated traits was very close to its single-trait predictive ability. A similar result was found by Fernandes et al. (2018)^161^ in sorghum where they reported the indirect prediction accuracy for biomass yield by plant height was similar to its single-trait and multi-trait prediction accuracies. Our findings will help breeders to reduce workload by performing indirect selection for expensive or labor-intensive focal traits by phenotyping early expressed correlated simple-to-measure traits at an early stage of the breeding pipeline.

## Conclusion

In this study, a rigorous investigation of various key factors affecting genomic predictive ability was conducted by comparing fourteen different models. The results indicated that models have a significant effect on predictive ability and no single model worked best across all traits, though model RR shows the potentiality by yielding higher predictive ability values for most of the trait. It is better to compare various models to choose the best one for any trait. Predictive ability reaches a plateau around a certain marker density and shows similarity with the whole marker set when choosing markers covering all QTL information. In the diverse flax collection used for this study, the small sample size representing population structure was strong enough to boost predictive ability compared to the whole collection across all traits. Along with this, indirect selection for seed yield considering correlated traits also holds potential for applied breeding efforts. This research presented herein will equip the plant breeders to efficiently design various aspects of genomic prediction to gain increased selection accuracy, which will subsequently accelerate the program’s genetic gain.

### Supplementary Information

Supplementary Legends.

Supplementary Figure S1.

Supplementary Figure S2.

Supplementary Figure S3.

Supplementary Figure S4.

Supplementary Figure S5.

Supplementary Figure S6.

Supplementary Figure S7.

Supplementary Table S1.

Supplementary Table S2.

Supplementary Table S3.

Supplementary Table S4.

Supplementary Table S5.

## Data Availability

All raw sequence data and variant data are available in the NCBI and EVA repositories. The accession IDs for them are PRJNA979944 (https://www.ncbi.nlm.nih.gov/sra/PRJNA979944) and PRJEB62432 (https://www.ebi.ac.uk/eva/?eva-study=PRJEB62432), respectively. The Phenotypic datasets and R scripts used and/or analyzed during the current study are available from the corresponding author upon reasonable request.
