# Supplementary material for: Genomic prediction for agronomic traits in a diverse Flax (Linum usitatissimum L.) germplasm collection
Source: Sci Rep. 2024 Feb 8;14:3196. doi: 10.1038/s41598-024-53462-w (PMC10850546; doi:10.1038/s41598-024-53462-w)
Supplement: Supplementary file 1 — Supplementary Legends. [file 41598_2024_53462_MOESM1_ESM.docx]

**Supplementary Tables**

Table S1. Details of genotypes utilized in this study with their clustering pattern.

Table S2. Phenotypic variability of different traits under different environments.

Table S3. Marker numbers for different scenarios are used to assess prediction ability.

Table S4: Variation in predictive ability values across traits according to marker subset based on chromosome-wise LD decay distance and random selection.

Table S5: Variation in predictive ability values across traits according to marker subset selected based on marker-trait association.

**Supplementary Figures**

Supplementary Figure S1. Field layout showing diagonal placement of check variety OME (Omega), GD (Gold ND) and NH (ND Hammond). The f denotes the target genotypes. Details of genotypes are available in the Supplementary Table S1.

Supplementary Figure S2. Frequency distribution of 337 genotypes across environments for 10 agronomic traits.

Supplementary Figure S3. Correlation among traits within environments.

E1 to E5 denotes different environments. Correlation_combined was calculated using all row data within five environments for all traits. DF is days to flowering, PH is plant height, TL is technical length, BN is branch number, BollN is boll number, TSW is thousand seed weight, SA is seed area, SW is seed width and SL is seed length. Color darkness increases proportionately with the increment of correlation-coefficient values.

Supplementary Figure S4. Correlation among environments in terms of traits.

Color darkness increases proportionately with the increment of correlation-coefficient values.

Supplementary Figure S5. Clustering of the whole collection using 26,171 SNP markers in STRUCTURE v.2.3.4. Estimated population structure of 337 genotypes on K = 5 using four alternative statistics ^77^.

Supplementary Figure S6. LD decay pattern of 15 chromosomes of flax.

Supplementary Figure S7. Boxplot of predictive ability values across models for 8 agronomic traits. SVM, LASSO and EN model was not shown due to their low predictive ability. PH is plant height, TL is technical length, BN is branch number, BollN is boll number, TSW is thousand seed weight, SA is seed area, SW is seed width and SL is seed length.
