# Supplementary figures and images for: Genomic prediction for agronomic traits in a diverse Flax (Linum usitatissimum L.) germplasm collection
Source: Sci Rep. 2024 Feb 8;14:3196. doi: 10.1038/s41598-024-53462-w (PMC10850546; doi:10.1038/s41598-024-53462-w)

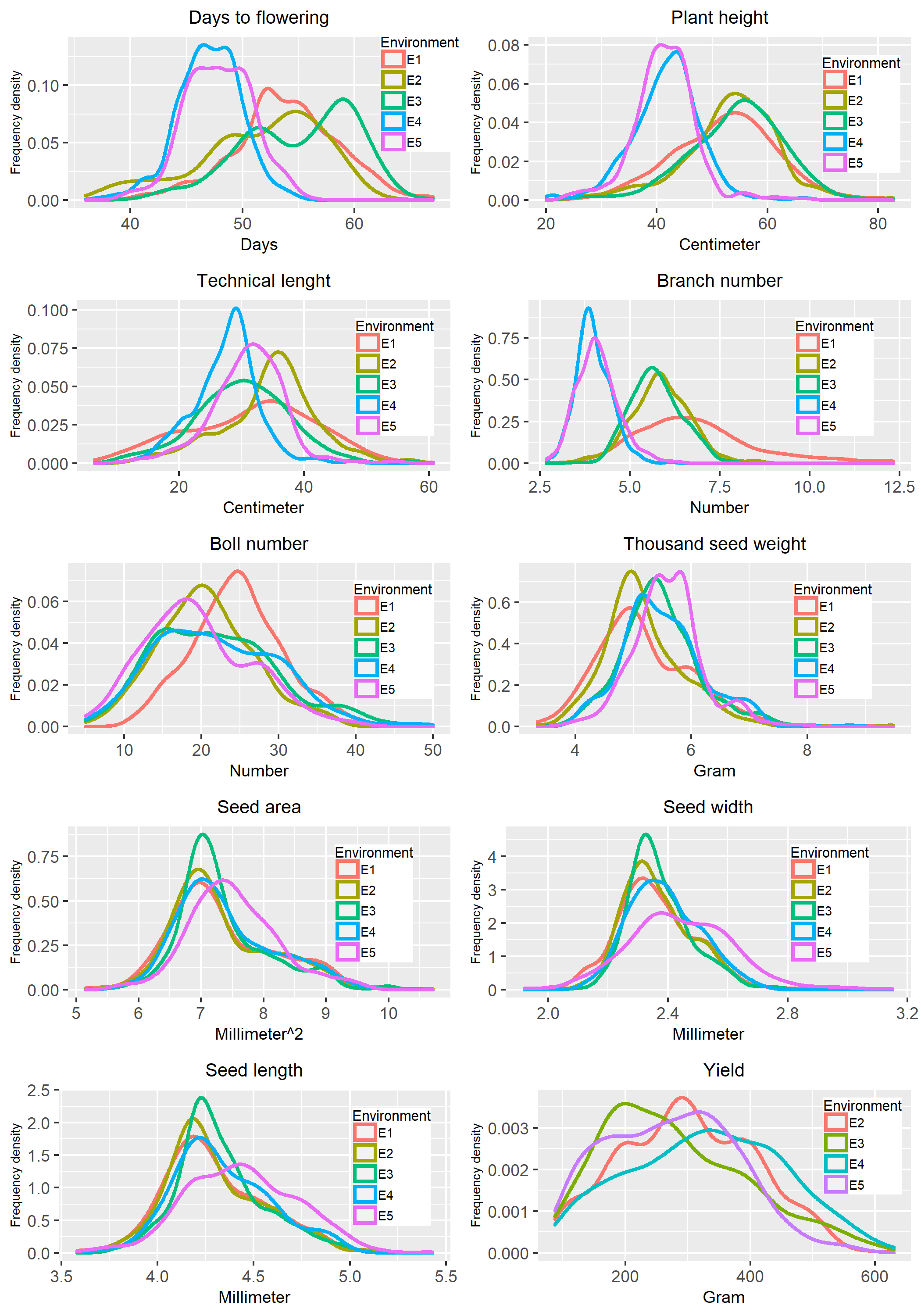

Supplement: Supplementary file 3 — Supplementary Figure S2. [file 41598_2024_53462_MOESM3_ESM.tiff]

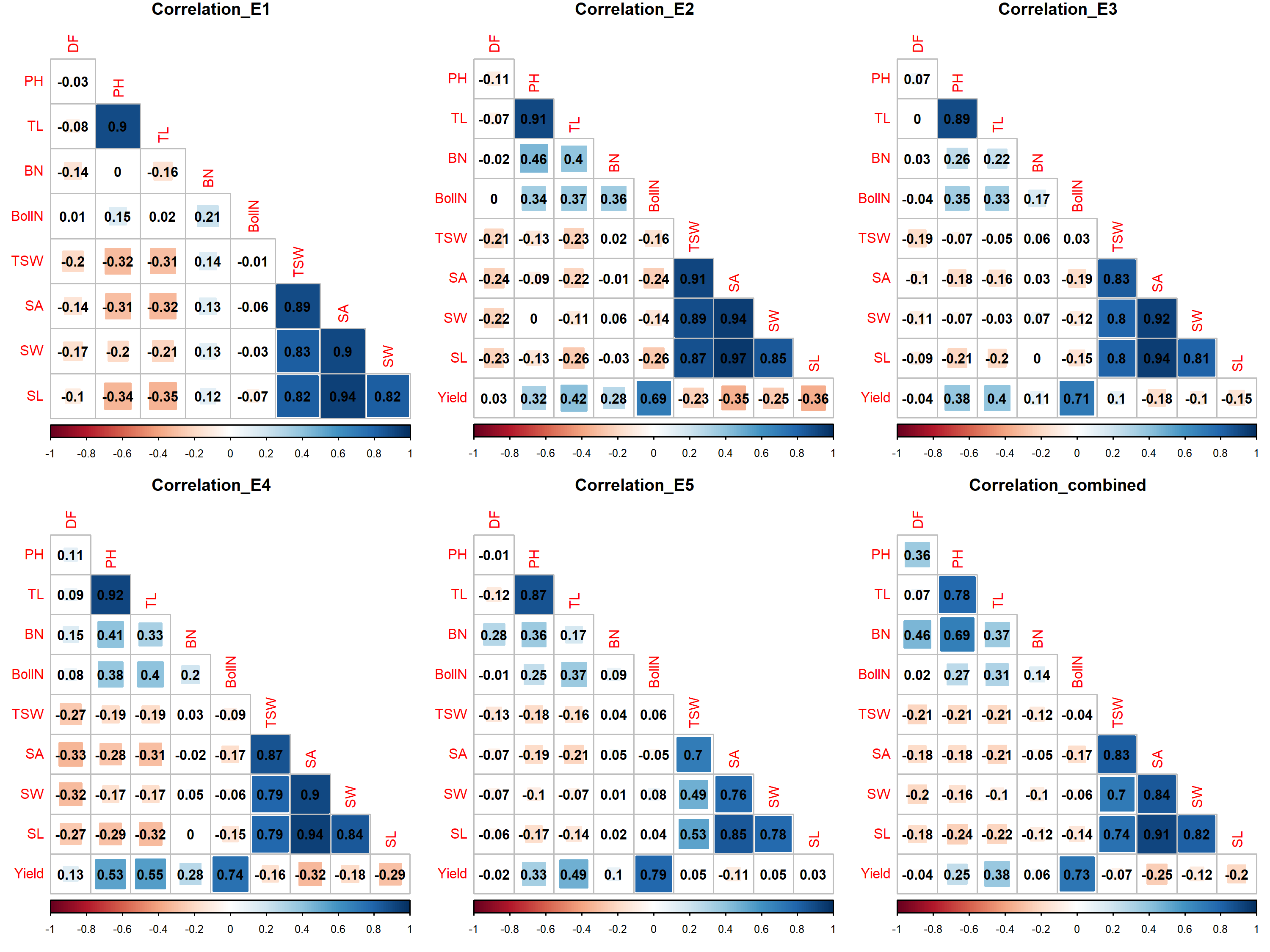

Supplement: Supplementary file 4 — Supplementary Figure S3. [file 41598_2024_53462_MOESM4_ESM.tiff]

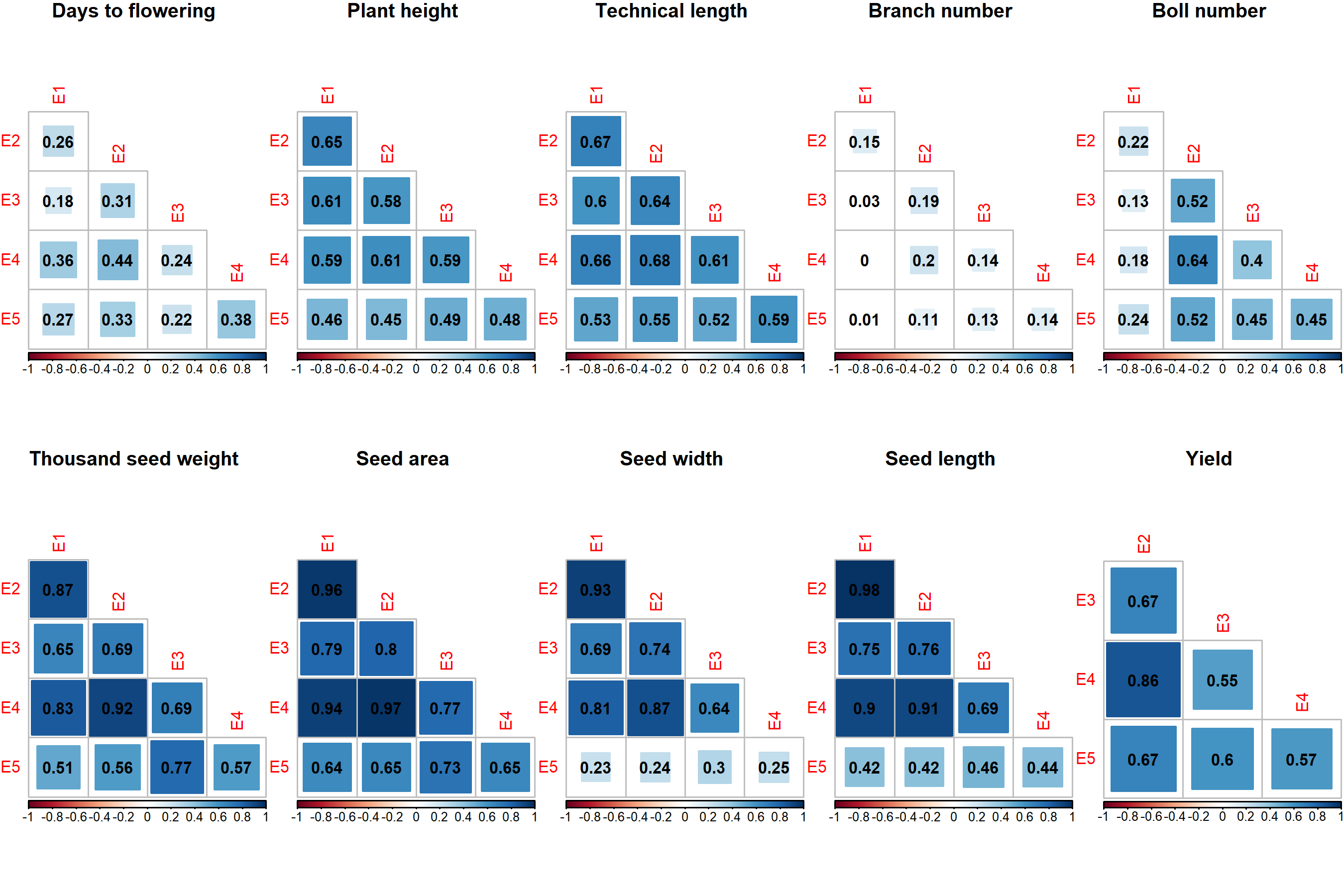

Supplement: Supplementary file 5 — Supplementary Figure S4. [file 41598_2024_53462_MOESM5_ESM.tiff]

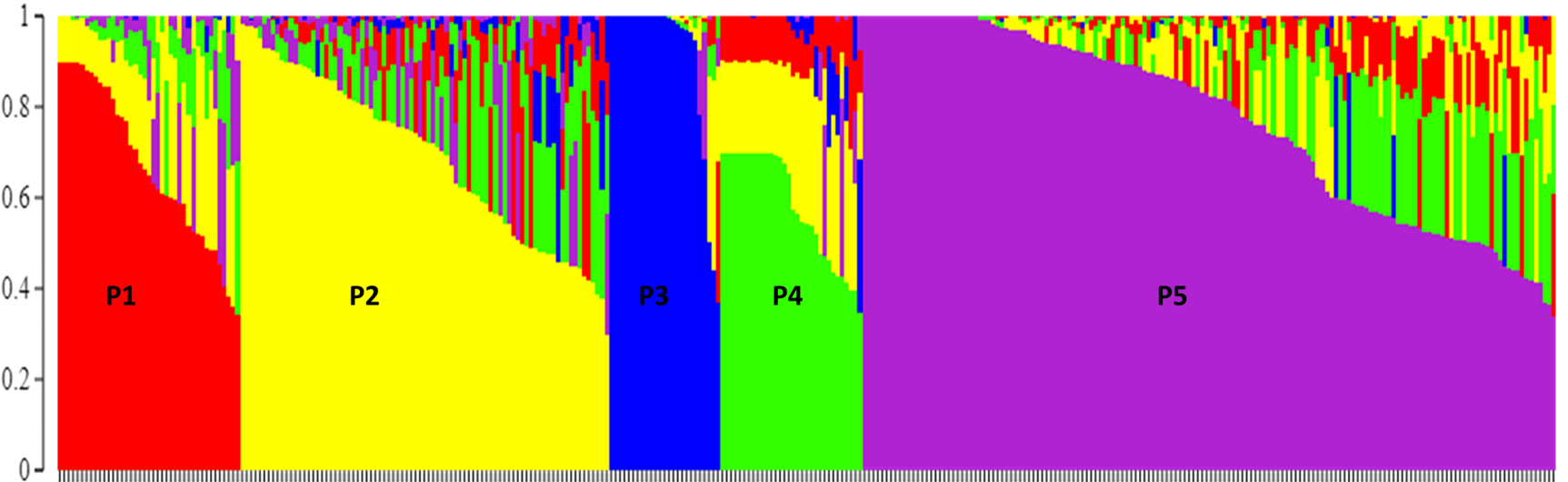

Supplement: Supplementary file 6 — Supplementary Figure S5. [file 41598_2024_53462_MOESM6_ESM.png]

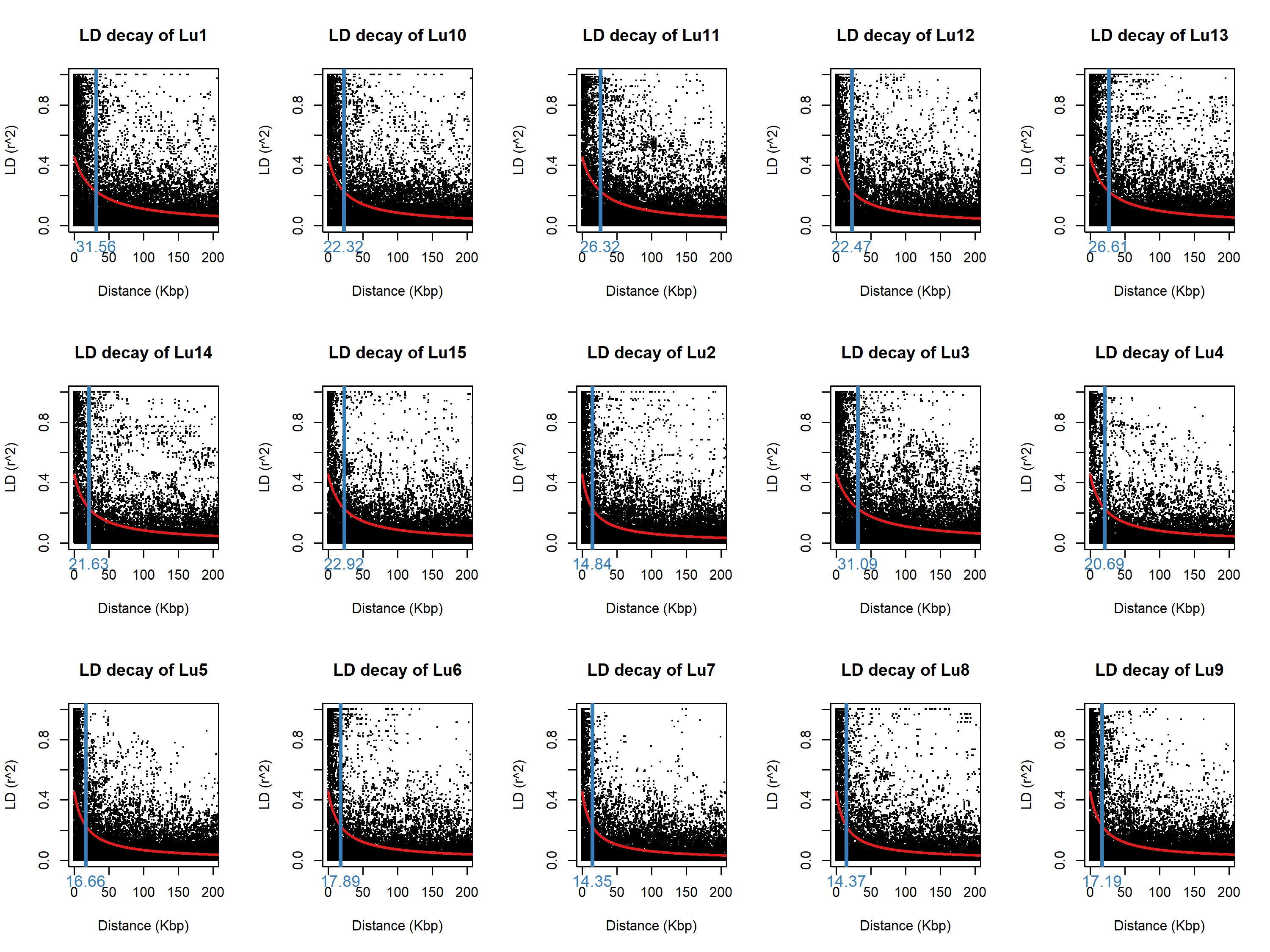

Supplement: Supplementary file 7 — Supplementary Figure S6. [file 41598_2024_53462_MOESM7_ESM.tiff]
